# Wide-dynamic-range promoters engineered for cyanobacteria

**DOI:** 10.1186/1754-1611-7-10

**Published:** 2013-04-22

**Authors:** Hsin-Ho Huang, Peter Lindblad

**Affiliations:** 1 Microbial Chemistry, Department of Chemistry - Ångström Laboratory, Uppsala University, P.O. Box 523, SE-75120, Uppsala, Sweden

## Abstract

**Background:**

Cyanobacteria, prokaryotic cells with oxygenic photosynthesis, are excellent bioengineering targets to convert solar energy into solar fuels. Tremendous genetic engineering approaches and tools have been and still are being developed for prokaryotes. However, the progress for cyanobacteria is far behind with a specific lack of non-native inducible promoters.

**Results:**

We report the development of engineered TetR-regulated promoters with a wide dynamic range of transcriptional regulation. An optimal 239 (±16) fold induction in darkness (white-light-activated heterotrophic growth, 24 h) and an optimal 290 (±93) fold induction in red light (photoautotrophic growth, 48 h) were observed with the L03 promoter in cells of the unicellular cyanobacterium *Synechocystis* sp. strain ATCC27184 (i.e. glucose-tolerant *Synechocystis* sp. strain PCC 6803). By altering only few bases of the promoter in the narrow region between the -10 element and transcription start site significant changes in the promoter strengths, and consequently in the range of regulations, were observed.

**Conclusions:**

The non-native inducible promoters developed in the present study are ready to be used to further explore the notion of custom designed cyanobacterial cells in the complementary frameworks of metabolic engineering and synthetic biology.

## Background

Promoter studies in the unicellular cyanobacterium *Synechocystis* sp. strain PCC 6803 (*Synechocystis*) mainly focus on elucidating their native transcriptional regulations to different environmental stimuli and changes. For example, some promoters are light responsive (*psa*[1], *psb*[2], and *secA*[3]), dark-inducible (*lrtA*[4]), nitrate/nitrite-inducible (*nirA*[5]), copper-ions responsive (*petE*[6]), and heavy metal-ions inducible promoters [7]. The promoters can be categorized into three different types (I, II, and III [8]) depending on how the binding motifs arranged to interact with the respective sigma factor and cognate transcription factor. Type I promoters fit the eubacterial canonical promoters [9] with the -35 and -10 elements being spaced by 17-bp. Type II promoters, with the -10 element only or including an enhancer-motif for binding an activator protein, are in majority. Type III promoters are distinct from type I and II for stringent responses. In the framework of synthetic biology, the implemented transcription regulatory system is expected to have minimal cross-talks with the host’s genetic background [10]. Therefore, it is necessary to develop non-native promoters regulated by foreign transcription factor and non-metabolized ligands.

Until today, the number of non-native promoters examined in *Synechocystis* is very limited, e.g. the λP_L_-derived BBa_R0040, λP_R_-derived BBa_R0051, and P_*lac*_-derived BBa_R0010 promoters in the BioBrick Registry (http://partsregistry.org/Promoters), and the P_*trc*_ promoter [11]. The three former promoters, although characterized in *Escherichia coli*, do not function in *Synechocystis*. A leaky repressed activity and a narrow range of regulation are the common problems of the strong P_*trc*_ promoter in the unicellular cyanobacteria *Synechocystis*[11] and *Synechococcus*[12,13]. The most commonly used systems to express and over-express genes are based on LacI-regulated *trc* and *tac* promoters [12-16]. The lack and limitations of available promoters for cyanobacterial biotechnology initiated our present work to develop non-native promoters that are fully repressed and highly induced. The regulatory system used is based on the foreign transcription factor TetR repressor and its ligand inducer anhydrotetracycline (aTc) [17].

Transcription initiation is a key point for regulating the gene expression [18]. Five promoter elements such as the UP element, -35 element, extended -10 element, the -10 element, and a nucleotide in two positions downstream of the -10 element are essential for the interactions with RNA polymerase (RNAP) [9]. RNAP is the key enzyme performing transcription in three stages: initiation, elongation, and termination [19]. One way to regulate transcription initiation is to repress it when a transcription factor binds to its cognate site. The transcription factor’s binding creates a steric hindrance usually close to the essential promoter elements to prevent RNAP’s binding to the promoter. The λP_L_-derived BBa_R0040 promoter, termed the R40 promoter in the present work, serves as a template for promoter engineering. The promoter region to keep, covering the first three essential promoter elements, contains the two TetR repressor’s binding sites with the consensus -35 element in between. The promoter region to engineer is the -10 element and the indicated nucleotides in the region between the -10 element and the transcription start site. We created a L promoter library (denoting its origin from the λP_L_ promoter [20]) to be examined in *Synechocystis*.

It is known from the studies of *Escherichia coli*[21,22] and *Thermus aquaticus*[23,24], but with no information for cyanobacteria, that the 5′-GGG-3′ located immediately downstream of the -10 element on the non-template strand of promoter DNA is crucial for transcriptional regulation and promoter strength, especially the G in the second position. In the present work we systematically change the nucleotide sequence in this region on promoter, examine its strength, and demonstrate the first functional TetR-regulated promoter library in the cyanobacterium *Synechocystis*. The potential applications of this library are discussed.

## Results

### Altering the -10 element and its downstream sequence makes promoters function in *Synechocystis*

**Figure 1:**
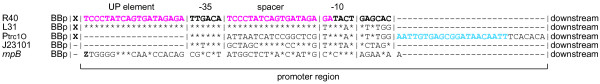
**Alignment of selected promoters and their flanking sequences. **The reporter construct of R40 promoter (i.e. BBa_R0040) is used as a reference sequence; gap (hyphen ‘-’) and identical base (asterisk ‘*’) are indicated for the each promoter. ‘BBp’ is the BioBrick prefix (GAATTCGCGGCCGCTTCTAGAG); ‘X’ is the TetR repressor expressing device [BBa_J23101]-[BBa_P0440] and the 8-bp BioBrick scar (TACTAGAG); ‘downstream’ sequence to the initial codon ATG of the reporter EYFP gene are the 8-bp BioBrick scar, the 10-bp ribosome binding site RBS*, the 6-bp BioBrick scar, and the 3-bp initial codon (TACTAGAG|TAGTGGAGGT|TACTAG|ATG); the *tetO2 *operator of the Tn*10 *resistance operon was placed upstream of the -35 element and in the spacer region for the two TetR-binding sites (bases shown in magenta). Core bases of each operator for the TetR binding are underlined. The R40-derived promoters L01 to L16, L21 and L22 are compared in Table 1. The *lacO1* operator of the P_trc1O _promoter contains the bases shown in cyan. The *rnpB *promoter is aligned with the -10 element CACACT [25] and Z is the remaining 153 bases.

To overcome the very low R40 promoter strength problem previously identified in *Synechocystis*, contrary to its high strength in *E. coli*[11], the L12 promoter was created by changing the -10 element of R40 promoter with the consensus TATAAT of *Synechocystis*[25]. In the presence of the TetR repressor and with the design of two TetR-binding sites on either R40 or L12 promoter (Figure 1), the regulation of a EYFP gene expression driven by either promoter was observed under both an induced and a repressed condition, in the presence and absence of the TetR’s inducer aTc, respectively. As result (Table 1), in both induced and repressed conditions, the promoter strength of L12 is lower than the one of R40. Similarly, the L12 promoter’s induction fold, which is the ratio of promoter strength in the induced to the repressed condition, is also lower than observed for R40’s.

**Table 1:** Promoter comparisons in sequence, regulation, induction and thermal opening probability pattern

**Promoter**	**-10 element and its downstream 8 bases**^**a**^	**Regulation**^**b**^		**Induction**^**c**^	**Pattern**^**d**^
		**Induced**	**Repressed**		
L15	TATAATG**GA**CACT	20.1 ± 0.1	0.243 ± 0.003	79 ± 1	A 〈17.9 ± 0.2〉
L07	TATAATG**GT**CACT	19.8 ± 0.1	0.289 ± 0.003	65 ± 1	
L05	TATAATG**CT**CACT	16.3 ± 0.1	0.198 ± 0.003	78 ± 1	
L13	TATAATG**CA**CACT	15.5 ± 0.1	0.219 ± 0.003	67 ± 1	
L03	TATAATG**GC**CACT	19.2 ± 0.1	0.220 ± 0.003	83 ± 1	B 〈16.3 ± 0.2〉
L11	TATAATG**GG**CACT	19.1 ± 0.1	0.236 ± 0.003	77 ± 1	
L09	TATAATG**CG**CACT	15.6 ± 0.1	2.88 ± 0.01	5.15 ± 0.01	
noLVA_L09^e^	TATAATG**CG**CACT	11.53 ± 0.05	0.545 ± 0.004	20.1 ± 0.1	
L01	TATAATG**CC**CACT	11.30 ± 0.05	0.235 ± 0.003	46 ± 1	
L02	TATAATG**TC**CACT	17.7 ± 0.1	0.214 ± 0.003	79 ± 1	C 〈15.9 ± 0.2〉
L10	TATAATG**TG**CACT	17.6 ± 0.1	0.235 ± 0.003	71 ± 1	
L04	TATAATG**AC**CACT	12.3 ± 0.1	0.201 ± 0.003	58 ± 1	
L12	TATAATG**AG**CACT	0.043 ± 0.003	0.022 ± 0.003	1.9 ± 0.3	
L16	TATAATG**AA**CACT	17.5 ± 0.1	0.240 ± 0.003	69 ± 1	D 〈15.1 ± 0.2〉
L06	TATAATG**TT**CACT	15.8 ± 0.1	0.219 ± 0.03	69 ± 1	
L08	TATAATG**AT**CACT	14.9 ± 0.1	0.255 ± 0.003	56 ± 1	
L14	TATAATG**TA**CACT	12.1 ± 0.1	0.304 ± 0.003	38.0 ± 0.4	
L21	TATAAT ***GGGA*** GCT	41.6 ± 0.2	40.1 ± 0.2	0.986 ± 0.003	E
L22	GATACT ***GGGA*** GCT	0.378 ± 0.003	0.023 ± 0.004	16 ± 2	F
P_trc1O_	TATAATGTGTGA	46.4 ± 0.2	43.9 ± 0.2	1.007 ± 0.003	G
L31	TATAATGTGTGGT	3.08 ± 0.01	0.169 ± 0.003	17.3 ± 0.3	H
R40	GATACTGAGCACT	0.272 ± 0.003	0.082 ± 0.003	3.2 ± 0.1	I
J23101	TATTATGCTAGCTA	4.57 ± 0.02	4.461 ± 0.02	0.974 ± 0.003	n.s.
*rnpB*	CACACTAGAAAAAT	1.00 ± 0.01 (427 ± 2)	1.00 ± 0.01 (448 ± 2)	0.95 ± 0.01	

^a^, The sequences of listed promoters, excluding P_trc1O_*,* J23101, and *rnpB *promoters, are identical except the region shown in this table. The consensus -10 element TATAAT is underlined. The transcription start site (TSS) is boxed. The promoter sequences are detailed (Figure 1). ^b^, The induced and repressed conditions are *Synechocystis *sp. strain ATCC27184 (i.e. glucose-tolerant *Synechocystis* sp. strain PCC 6803) cells in LAHG growth condition treated with and without 100 ng/mL aTc, respectively for 24 hours. The mean ± standard error of mean (s.e.m.) is relative to the strength of the *rnpB *promoter in the respective regulation condition. The value in parentheses is the experimental mean ± s.e.m. of EYFP emission per cell after subtracting the auto-fluorescence of *Synechocystis* cells containing pPMQAK1 vector only. The measurement is done by a flow cytometer to collect 50,000 events for each biological sample. ^c^, The induction of a promoter is the ratio of its measured strength in induced compared to in repressed condition. ^d^, The simulated thermal opening probability patterns (A-I) at 303 K are shown (Figure 4); n.s., not simulated. The value in a bracket is the mean ± s.e.m. under the induced condition of strengths of the promoters in a given pattern. The noLVA_L09 and L12 constructs are excluded in averaging. ^e^, The only difference of noLVA_L09 to L09 is the introduction of a double stop codon in the end of the *tetR* gene to cease translation of a protease LVA tag tailing in C-terminal of TetR repressor. The L09 promoter regions of both are identical.

To further address the challenge of an even lower strength of the L12 promoter whose design fits a canonical promoter for eubacteria [9], we hypothesized that replacing bases in the region between the -10 element and the transcription start site (TSS) of a promoter can increase the L12 promoter strength. This region has been proved to significantly influence the promoter strength [21] but no information is available for cyanobacteria. Three parallel modifications in this region were designed (Table 1): the first line, the position(s) locating on either one or both of the second and third bases immediately downstream of -10 element of L12 promoter was/were varied with adenine or guanine or thymine or cytosine and in total 16 promoters, from L01 to L16, were constructed; the second line, a *bona fide* downstream basal promoter element GGGAgc [24] was designed immediately downstream of the -10 element of L12 and R40 promoter as L21 and L22 promoters, respectively; the third line, a -10 element downstream sequence, GTGTGG, of a strong P_trc1O_ promoter [11] was replaced in the same position of the L12 promoter to generate the L31 promoter. J23101 and *rnpB* promoters were used as references when comparing promoter strength [11].

We first observed that, in the induced condition, replacing with the consensus -10 element may affect promoter strength positively or negatively depending on its adjacent downstream sequence (Table 1). It increases the L21 promoter strength by 110 (±1) -fold compared to L22 when a *bona fide* downstream basal promoter element GGGA exists; however, on the contrary, it decreases L12 promoter strength by 6 (±0.4) fold compared to R40 when its adjacent downstream sequence, G**AG**CACT, exists. To further gain insight of this region between -10 element and TSS of native type I promoters [8] of *Synechocystis*, the sequence conservation weighted by the number of pyrosequencing-reads from differential RNA-sequencing data [25] is G**A**GGG adjacent to -10 element; adenine locating 2 nt downstream from the -10 element is locally more conserved than other bases as revealed by the weighted sequence logo (Additional file 1: Figure S1). Together with the presence of the consensus -10 element TATAAT of *Synechocystis*, we argue that the even lower promoter strength of L12 compared to R40 might come from the deleterious effect of a fully consensus promoter [9].

We then confirmed our hypothesis to increase the L12 promoter strength in the induced condition (Table 1). First, in L01 to L16 promoters, by merely changing two bases, A and G, of L12, to G and A in L15 and C and C in L01 show up to 467 (±33) fold and at least 263 (±18) fold improvement in promoter strength, respectively. For the strong L15, L07, L03, and L11 promoters, they coincide with a G locating 2 nt downstream from the -10 element. Second, in the L21 and L22 promoters, a GGGA motif largely enhances promoter strength by 967 (±68) fold from L12 to L21, but only slightly increases promoter strength by 1.4 (±0.02) fold from R40 to L22 together with a non-consensus -10 element. The L15 and L21 are 20 (±0.2) and 42 (±0.5) times stronger than the *rnpB* promoter, respectively. Third, in the L31 promoter, the -10 element downstream sequence GTGTGG of the strong P_trc1O_ promoter increases its strength by 72-fold from the L12 promoter but the increased strength is still much lower than the P_trc1O_ promoter. Taking together, the deleterious effect of a fully conserved sequence on promoter strength was removed by replacing the conserved adenine and guanine at 2 and 3 nt, respectively, downstream of the consensus -10 element with any other three nucleotides. We further concluded that guanine locating 2 nt downstream from the consensus -10 element is essential for promoter strength.

### The L03 promoter is widely regulated in *Synechocystis*

The engineered L promoters are controlled by the transcriptional regulatory machinery composed of the aTc inducer, the TetR repressor, and the *tetO2* operator of the Tn*10* tetracycline resistance operon [17]. All promoters in the present study have two *tetO2* operators, except the P_trc1O_, J23101, and *rnpB* promoters with no *tetO2* operator; the core invert repeat in the operator for TetR binding is not affected after the change of the -10 element (Figure 1). The relative amount of TetR repressors in a cell of *Synechocystis* was balanced from its constitutive expression by the J23101 promoter and its more efficient turnover by being tagged by a protease tag, LVA [11].

In the absence of inducer aTc, each of the L01 to L16 promoters excluding the L12 and L09 showed very low but detectable repressed strength which was about 10-times higher than the repressed L12 promoter strength. The L09 promoter in the L09 construct showed the repressed strength about 100-times higher than the L12 promoter’s (Table 1). We hypothesize that increasing the TetR repressors by slowing down its turnover via the removal of a LVA tag from it can further repress the L09 promoter. As result, the L09 promoter can be further repressed in the noLVA_L09 construct than in the L09 construct, but the improved repressed L09 promoter strength is still 25-times higher than the L12 promoter’s.

**Figure 2:**
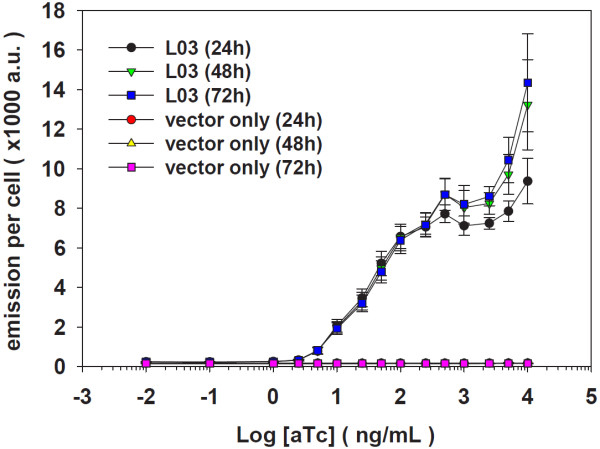
**Dose-dependence of the inducer anhydrotetracycline (aTc) on the induction of L03 promoter in cells of *Synechocystis *sp. strain ATCC27184 (i.e. glucose-tolerant *Synechocystis *sp. strain PCC 6803) under the LAHG growth condition. **The data were measured by flow cytometer to collect 50,000 events from each of three biological samples. The points in the plot represent mean ± s.e.m.a.u., arbitrary unit.

In the presence of the inducer aTc, a dynamic range of the transcription regulation on an L promoter is accessed with the induction fold: larger induction fold means wider dynamic range (Table 1). There is no dynamic range for the strong L21 promoter with two *tetO2* operators and for the promoters without *tetO2* operator such as P_trc1O_, J23101 and *rnpB* promoters. One way to expand the dynamic range is to further lower the repressed strength of a promoter: the L09 promoter gained about 4 (±0.02) times wider range in the noLVA_L09 construct than the L09 construct. But, increasing the repression by more TetR repressors compromised the induced promoter strength. Another way to expand the dynamic range is to increase the induced strength of a promoter: the L03 promoter acquired about 44 (±7) times wider range than the L12 promoter. But, the altered intrinsic properties derived from the mutated promoter sequence compromised the repressed state of the L03 promoter. Because of the L03 promoter showing widest dynamic range among the L promoters, the dose-dependence of inducer aTc was further determined between 1 and 10 μg/mL for this promoter. Also, the induction fold of this promoter can reach about 230 (±55) fold in 72 hours when cells of *Synechocystis* are in the light-activated heterotrophic growth (LAHG) mode and activated with white light (Figure 2). Therefore, we propose that the L03 promoter is widely regulated in *Synechocystis*.

### The regulation of the L03 promoter in *Synechocystis* under different physiological conditions

**Figure 3:**
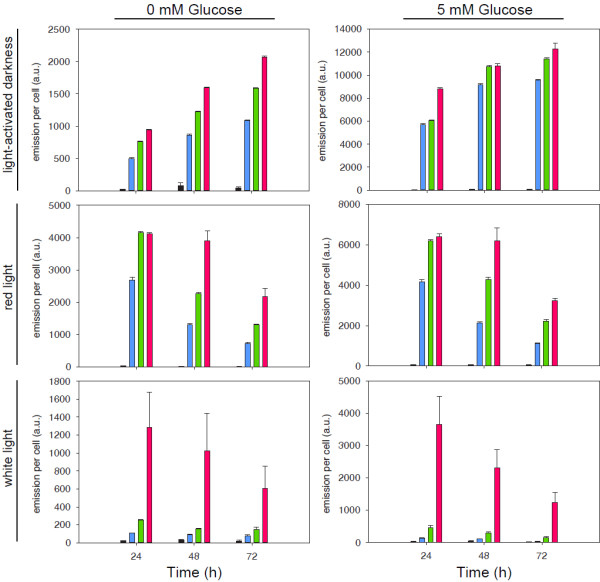
**The L03 promoter strength measured in *Synechocystis *sp. strain ATCC27184 (i.e. glucose-tolerant *Synechocystis *sp. strain PCC 6803) cells. **Cells were sampled on 24, 48, and 72 hours after induced with 0 (black bar), 10^2 ^(blue bar), 10^3^ (green bar), and 10^4 ^(magenta bar) ng/mL aTc and grown under different light conditions: light-activated darkness (upper panel), red light (middle panel), and white light (lower panel). The *Synechocystis *cells were grown in kanamycin-added BG11 medium supplemented with 0 mM (left panel) or 5 mM (right panel) glucose. The EYFP emission per cell has been subtracted with auto-fluorescence of a cell containing pPMQAK1 plasmid only. a.u., arbitrary unit. The detail induction data can be found in Additional file 1: Table S2.

To examine how the environmental conditions affect the regulation of L03 promoter in cells of *Synechocystis*, the combinations of two glucose concentrations (0 and 5 mM), four aTc concentrations (0,10^2^,10^3^,10^4^ ng/mL), three light conditions (darkness, 20 μmol m^-2^ s^-2^ red light, and 30 μmol m^-2^ s^-2^ white light) were varied and the data collected on three time points (24, 48, and 72 hours after treatment) (Figure 3). On a time point in a light condition, in common, EYFP expression level is higher when induced by a higher aTc concentration in the presence of glucose. However, when treated with the consistent glucose and aTc concentrations, the trend of EYFP expression with time is increasing in darkness but is decreasing under light. Furthermore, the trend decreases faster in white light than red light. This is due to the photolability of aTc and the overlapping of aTc absorption spectrum with the spectrum of photons emitted from white LEDs (Additional file 1: Figure S2). The photolability of the inducer aTc does not diminish the significance of functional L promoters for photosynthetic microorganisms. In the present study, the inducer serves as an easy-to-use ligand to develop TetR-regulated L promoters. Depending on application and purpose alternative molecules, not using aTc, to induce TetR-regulated L promoters under light are proposed in the discussion. To conclude, for the L03 promoter, the best induction in darkness is 239 (±16) fold in 24 hours under the LAHG condition with white light and the best induction in red light is 290 (±93) fold in 48 hours under photoautotrophic condition when treated with 10 μg/mL aTc. 

### The spontaneous thermal openings of an engineered promoter simulated by the PBD model

**Figure 4:**
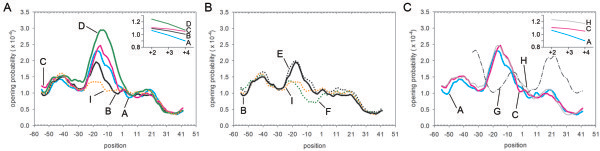
**Spontaneous thermal opening probability simulated at 303 K by the average stacking PBD model **[26]**of a promoter sequence. **(**A**) The pattern I (orange dot line) of the R40 promoter is compared to the four patterns, A, B, C and D (cyan line, black line, magenta line, and green line, respectively) simulated from 16 R40 -derived promoters (from L01 to L16). The grouping is shown (Table 1). (**B**) The pattern E (black dot line) and F (green dot line) of L21 and L22 promoters, respectively, are compared to the pattern B (black line) and the pattern I (orange dot line). (**C**) The patter G (black dash-dot line) and H (gray line) of P_trc1O _and L31 promoters, respectively, are compared to pattern A (cyan line) and C (magenta line). Position +1 is the TSS of a given promoter. The opening probability at a position is read as the probability of a base pair to open at that position and its consecutive downstream 5 base pairs with the amplitude larger than 2.8 Å.

To understand how the thermal opening probability of a pre-melting DNA strand changes after the substitution of bases on indicated positions of a promoter, this was analyzed theoretically with the well-established Peyrard-Bishop-Dauxois (PBD) model via a Monte Carlo method [26]. Due to the sequence-independent stacking term using an averaged constant in this model, 16 L promoters can be only analyzed in four patterns categorized by hydrogen bonds of a modified base pair (Table 1). The simulation was successfully repeated in the present study at 303 K by showing the correspondence between the opening probability peak and the cognate site of a transcriptional regulator on corresponding positions: the two *tetO2-*operator cores (Figure 1) at the respective regions from -52 and -40 and from -27 to -15 (Figure 4A, 4B); the *lacO1* operator in the region from +2 to +22 (pattern G in Figure 4C).

For an L promoter, a locally higher opening probability peak was observed in the region between -27 and +3, where locates the core of *tetO2* operator in the spacer region, the -10 element, and a 5-nt region. But, for the P_trc1O_ promoter, the peak was observed in the region between -34 and -29, where locates the -35 element. For the R40 promoter, it showed a lack of a clear peak around the TSS. The consensus -10 element significantly increases the opening probability in the region between -27 to +3 when comparing the pattern C and I from the promoter L12 and R40 (Figure 4A) and when comparing pattern E and F from the promoter L21 and L22 (Figure 4B), respectively.

In the region between -13 and -4, the opening probability from high to low correlates with the order and number of hydrogen bonds as (2,2), (2,3), (3,2), and (3,3) of the substituted base pairs at position -6 and -5, respectively, when observing in the direction of strand separation [27] propagating from -10 element to TSS (Figure 4A). A thermal opening pattern of the promoters showing a higher averaged value in strength (Table 1) has lower opening probability in the region between +2 and +4 (Figure 4A). Similarly, the weak L31 promoter shows higher opening probability in this region when comparing to the pattern A and C of strong L promoters (Figure 4C). From the pattern G of the strong P_trc1O_ promoter, it showed higher opening probability in the flanking region of the -35 element but not of the -10 element (Figure 4C).

### Evaluation of the engineered L promoters

**Figure 5:**
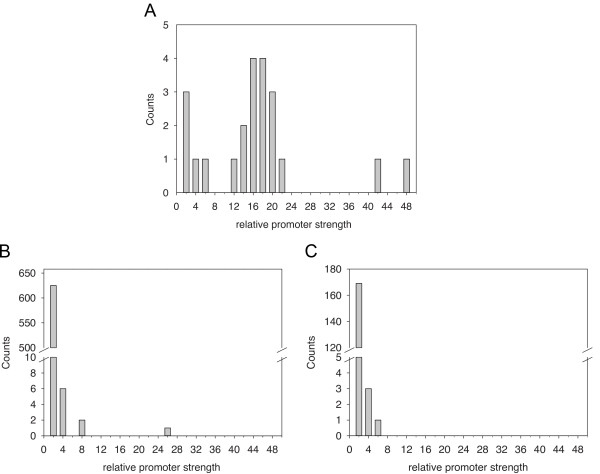
**Evaluation of the engineered promoters (A) and the native promoters (B and C) by the relative promoter strength in *Synechocystis *PCC 6803 under the respective growth condition. **(**A**) The histogram of the relative promoter strength by comparing 22 engineered promoters to the native *rnpB *promoter. The original data in EYFP emission of EYFP reporter proteins per cell are from the present work. (**B**) The histogram of the relative promoter strength by comparing 634 native type I promoters to the native non-type-I *rnpB *promoter. The original data in pyrosequencing-reads of RNA transcripts are from the supplementary Table three in the work of Mitschke *et al. *[25]. (**C**) The histogram of the relative promoter strength by comparing 173 native promoters, which transcribe protein-coding genes in high level, to the native *rnpB *promoter. The original data in pyrosequencing-reads of RNA transcripts are from the supplementary Table five in the work of Mitschke *et al. *[25].

Promoter strength is accessed by the amount of transcripts transcribed by a promoter: more transcripts mean a stronger promoter and *vice versa*. In the present work, the transcript levels are measured indirectly from the emission level of the fluorescent protein EYFP. Due to the standardized design with identical transcriptional terminators, ribosome binding sites, and stop codons arranged uniformly on the plasmid, the ratio of emission data may represent the ratio of transcript levels when comparing an engineered L promoter to the *rnpB* promoter (i.e. the relative promoter strength) under the LAHG growth condition (Table 1). The corresponding histogram of the relative promoter strengths is shown in Figure 5A. Similarly, using original data in a recent publication by Mitschke *et al.*[25] for native type I promoters and native promoters of the protein-coding genes in cells grown under photoautotrophic condition are shown in Figure 5B and Figure 5C, respectively. In the respective growth condition, the engineered L promoters, after induction, showed wider distribution in the relative promoter strength while the native promoters centered around 2-fold relative promoter strength. Interestingly, the native *rnpB* promoter, known to be unaffected by e.g. light/dark transitions, and redox perturbation [28-30], showed a consistent detection-threshold-to-promoter-strength ratio around 0.024 (±0.05) from the two totally different methods (present study and [25]). In addition, the analysis strongly indicates fully repressed states in both the L12 and L22 promoters. However, the L22 promoter has considerable range of induction whereas the L12 promoter does not (Table 1).

## Discussion

In the transcriptional initiation, a pre-melting promoter DNA undergoes sequential intermediate steps with the RNA polymerase (RNAP) to form a stable open complex; RNAP binding, formation of close complex RP_c_, formation of the bent and wrapped close complex I_1_, formation of initial open complex I_2_, and, finally formation of a stable open complex RP_o_[9,31]. After the formation of RP_o_, the RNAP proceeds to the transcription initiation through the DNA scrunching mechanism in which an obligatory stress intermediate forming as an extra unwinding DNA has been proposed to provide the driving force in promoter escape [32]. The abortive/productive ratio of transcription initiation may be influenced by the three competitive pathways; abortive cycle, scrunching pathway, and promoter escape–analyzed in the kinetic model of transcription initiation [33]. The RNAP escapes the promoter and enters the transcription elongation and termination [19]. In the present work, the process after the promoter escape should both be standardized in respect to the design of the construct and be well-controlled during the experiment. Therefore, the effect of the modified promoter sequence on the intrinsic thermal properties of its pre-melting state and on the productive transcriptional initiation is discussed by the simulated spontaneous thermal opening probability and the measured promoter strength, respectively.

Changing to the consensus -10 element TATAAT of *Synechocystis* clearly elevates the spontaneous thermal opening probability in the region between -27 and +3 (Figure 4B) and increases promoter strength significantly (Table 1) when comparing the L21 to the L22 promoter. A pre-melting promoter DNA with the elevated spontaneous opening probability may be more prone to expose the cognate bases for contacting with a DNA-binding protein such as σ subunit of RNAP and thus to increase the binding probability [26]. The recognition of the -10 element by σ subunit of RNAP has been proposed through the capture of two highly-conserved flipped-out bases and the extensive contact with the sugar-phosphate backbone of each nucleotide in the -10 element on the non-template strand; the recognition is coupled to strand separation and is the key step in initiating transcription [27]. Therefore, the increased L21 promoter strength may be due to a more favourable σ subunit binding compared to the L22 promoter. This is supported by similar result when comparing the mutated *lac* UV5 promoter with the wide-type *lac* promoter [26].

Changing to the consensus -10 element increases the L12 promoter’s opening probability (Figure 4A) but reduces its promoter strength (Table 1) when compared to the R40 promoter. The RNAP binding on the L12 promoter might be still enhanced based on the simulated result but the transcription initiation after formation of the close complex probably was impeded [31]. Through systematic modifications in the region between the -10 element and TSS, according to our hypothesis, an essential guanine at the position 2 nt downstream from the -10 element of L15, L07, L03, and L11 promoters was observed and represents a novel discovery for cyanobacteria. Interestingly, the results are consistent with an *in vitro* study of λP_L_, λP_R_, *rrnB* P1 and *rrnB* P2 promoters with RNAP from *Escherichia coli* demonstrating that the base identity at this position affects the life time of the promoter-RNAP complex after formation of the close complex [21] through a non-optimal contact with the region 1.2 of the σ^70^ subunit [22]. During the formation of the open complex it has been also proposed that the base identity at this position may serve as a “check point” in the propagating process of strand separation toward the TSS [27]. During the DNA scrunching, the position at 2 nt downstream of the -10 element locates within the extra unwinding DNA turn; substituting the base identity at this position might in consequence affect the promoter escape. Taken together, we speculate that substituting the base identity in the region between the -10 element and the TSS has the effect in fine-tuning the binding probability of the pre-melting DNA to the RNAP and has effect in the propagation of strand separation, the stability of open complex, and the balance in the three competitive kinetic pathways during transcription initiation. Therefore, any removal of the deleterious effect of a fully consensus sequence causes the improved promoter strength in the engineered L promoters other than the L12 promoter in *Synechocystis.*

Our attempt to fully repress the L09 promoter by increasing TetR repressors with the noLVA_L09 construct did not succeed. Based on i) the approximate 30 bp distance between the modified bases and a *tetO2* operator upstream of the -35 element of the L09 promoter and that ii) only the repressed L09 promoter showed leakage in gene expression, we speculate that the leakage of the repressed L09 promoter was caused by a so called “long-range effect of flanking single SNPs” [34] on the *tetO2* operator.

Because the L03 promoter shows widest dynamic range, it was chosen to study the effect of the environmental conditions such as glucose and light quality/quantity on the TetR-regulation of an L promoter. The beneficial effect of glucose on gene expression controlled by the L03 promoter may result from the metabolic balance between the decreased metabolites for the Calvin cycle and the increased metabolites for the oxidative pentose phosphate pathway and glycolysis [35,36]. The light quality and light quantity affect the light-sensitive inducer aTc differently in the TetR-regulation on the L promoter. The regulationin terms of induction fold and duration time is best in darkness, followed by in red light, and in white light. Since white light exists naturally,using this inducer might limit the applications with L promoters in photosynthetic microorganisms. However, thanks to the versatile TetRsystem, the TetR repressor can be induced by e.g. an RNA aptamer [37] and the short peptide TIP [38], which should be light-insensitive molecules.

Based on our results, a simple feed-forward loop for a lasting induction of TetR repressor is proposed (Additional file 1: Figure S3). This regulation initially still needs aTc to trigger the induction of the tight TetR-regulated L22 promoter, but when aTc loses its effect to induce in white light, the induction of TetR repressor could be over-taken by e.g. the presence of the RNA aptamer or the short peptide TIP.

In order to expand the dynamic range of induction, the narrow dynamic range of the tightly regulated L22 promoter can be indirectly amplified using the T7 RNAP and its cognate T7 promoters [39,40]. To reverse the regulation on L promoter, the revTetR repressor recognizes the same cognate site *tetO2* as the TetR repressor and the role of aTc changes from inducer to co-repressor [41]. It is also possible to further incorporate other regulatory mechanisms on RNA level, such as the orthogonal riboswitch [42], genetic switchboard [43], or RNA processing [44], and on protein level such as the post-translation modifications [45] that may lead to the design and construction of a programmable genetic circuit in *Synechocystis*. The TetR-regulated promoter library developed for the cyanobacterium *Synechocystis* may be combined with a systematic promoter selection method [46] to further design and construct the genetic circuit for robust control in gene expression.

## Conclusions

The novel non-native, wide-dynamic-range promoters developed in the present work demonstrates a significant step forward towards versatile purpose-driven applications with cyanobacteria in the framework of synthetic biology and metabolic engineering.

## Methods

### Strain and growth condition

Cells of the unicellular cyanobacterium *Synechocystis* sp. strain ATCC27184 (i.e. glucose-tolerant *Synechocystis* sp*.* strain PCC 6803) were grown in BG11_0_ medium [47] supplemented with 18 mM sodium nitrate under light condition indicated and agitated with horizontal orbiting at 120 rpm. *Escherichia coli* DH5α grew in LB medium at 37°C and agitated with horizontal orbiting at 250 rpm. According to plasmid used, the final concentration of antibiotic in the medium was 50 μg/mL (kanamycin), 35 μg/mL (chloramphenicol), or 100 μg/mL (ampicillin).

### Construction of promoter variants by PCR

TetR-regulated promoter variants of R40 promoter (i.e. BBa_R0040) were prepared by a PCR-based method with the universal forward primer XhoI_f_pSB2K3 and a respective reverse primer as listed in Additional file 1: Table S1. The reverse primer contains bases of the new promoter and restriction sites for further cloning. The approximate 1 kb PCR product was amplified from the BBa_pSB2K3 plasmid containing only the R40 promoter located in between the BioBrick prefix and suffix by using Phusion^®^ Hot Start High-Fidelity DNA Polymerase (Finnzymes). After amplification, DNA polymerase was removed by GeneJET™ PCR Purification Kit and the purified PCR product was digested simultaneously with FastDigest^®^ DpnI, XbaI and PstI. The DpnI digestion degrades the methylated template DNA, and the XbaI and PstI digestion enables the new promoter to be further cloned. The restriction enzymes were removed again with GeneJET™ PCR Purification Kit. With no need to retrieve the generated ~ 80 bp XbaI-PstI-digested DNA fragments by gel electrophoresis, the new promoter can be further cloned directly into the reporter construct with subsequent “3A assembly” method (http://partsregistry.org/Help:Assembly).

### Construction of reporter and control constructs by BioBrick standard assembly and 3A assembly

The complete reporter construct [BBa_J23101]-[BBa_P0440]-[new promoter]-[RBS*]-[BBa_E0130] was prepared with high copy-number BioBrick plasmids and then cloned the whole construct into the low copy-number shuttle plasmid pPMQAK1. In step one, the TetR repressor expressing device [BBa_J23101]-[BBa_P0440] and the EYFP reporter expressing device without a promoter [RBS*]-[BBa_E0130] were prepared in parallel by the “standard assembly” method (http://partsregistry.org/Help:Assembly) and resulted in BBa_pSB1A2 plasmids. The ribosome binding site, RBS*, was previously developed in our lab [48]. In step two, to avoid potential cloning problems, the new promoter was first assembled in downstream of the TetR device with the “3A assembly” method resulting [BBa_J23101]-[BBa_P0440]-[new promoter] in the BBa_pSB1K3 plasmid. In step three, using the “3A assembly” method again, the complete reporter construct was finished in the BBa_pSB1C3 plasmid. Meanwhile, for making the control constructs, BBa_J23101, and *rnpB* promoter, respectively, were assembled in upstream of [RBS*]-[BBa_E0130] by the “3A assembly” method resulting in the BBa_pSB1C3 plasmid. In final step, the completed reporter and control constructs were cloned, respectively, into the pPMQAK1 with the restriction sites EcoRI and PstI.

### LVA tag removal from the TetR repressor

The TetR repressor encoded in BBa_P0440 is tailed with a protease tag LVA for rapid degradation. This LVA tag can be removed by site-directed mutagenesis [49] introducing a double stop codon TAATAA in the 3′-end of *tetR* gene. The primers for site-directed mutagenesis are listed in Additional file 1: Table S1.

### Tri-parental mating

The shuttle vector pPMQAK1 bearing different constructs was transferred into *Synechocystis* cells by tri-parental mating as described previously [11].

### Anhydrotetracycline (aTc) induction in different growth modes of *Synechocystis* cells

To reduce operating error, a master *Synechocystis* cell culture was inoculated to have an absorption of 0.03 at 750 nm measured with a Plate Chameleon V Microplate Reader (Hidex) in BG11 medium with kanamycin supplemented either with or without 5 mM glucose. The master culture was distributed in aliquot to 3.5 mL each in culture wells of a 6-well tissue culture plate (SARSTEDT, 1.83.1839). The plates were agitated with horizontal orbiting at 120 rpm under 30 μmol photons m^-2^ s^-1^ of white light for 24 hours before aTc induction. For aTc induction, 3.5 μL 1000-fold stock of aTc was respectively added to the 3.5 mL cultures to reach the following final concentrations (ng/mL): 10^-2^, 10^-1^, 10^0^, 2.5, 5, 10^1^, 2.5x 10^1^, 5x 10^1^, 10^2^, 2.5x 10^2^, 5x 10^2^, 10^3^, 2.5x 10^3^, 5x 10^3^, 10^4^, and 10^5^. Since the aTc stock was prepared in 50% (v/v) ethanol, for 0 ng/mL aTc group, 3.5 μL 50% ethanol was added into the cell culture. After aTc induction, the cultures were exposed to either 30 μmol photons m^-2^ s^-1^ of white light, 20 μmol photons m^-2^ s^-1^ of red light, or in darkness. The reduced photon density of the red light aims for less negative effect on the aTc stability compared to in white light. The light source is a home-built LED light panel: 12 modules arranged in a 2-by-6 array and each module arranging 9 LEDs evenly distributed above each well of a 6-well plate. The spectrum of a LED is shown (Additional file 1: Figure S2) and the photon density was measured using a light sensor (Skye instruments) placed between the LEDs and the surface of the cell cultures. The cultures under different light conditions were stacked on the same shaker for identical 120 rpm agitation. The cultures were sampled every 24 hours by withdrawing 50 μL culture volume and diluted it with 950 μL fresh medium for determining specific growth rate and subsequent flow cytometry analysis. When sampling every 24 hours under the room light, it provides the white-light-activation condition for the culture growing in darkness as the LAHG growth mode [50].

### Transcription start site determination

The total RNA in cells of *Synechocystis* was prepared by a PGTX-based method [51]. The transcription start site was determined by 5′ RACE System for Rapid Amplification of cDNA Ends kit (Invitrogen, 18374–058) using GSP1, GSP2 and nested GSP primers as detailed in Additional file 1: Table S1.

### Flow cytometry

The emission from reporter EYFP expressed in a single *Synechocystis* cell was measured by BD™ LSRII flow cytometer with BD FACSDiva™ Software. *Synechocystis* cells harboring pPMQAK1 only were used as negative control and cells bearing reporter construct of P_trc1O_ promoter were used as positive control. The cytometer settings were: 786 volt for FSC, 412 volt for SSC, 676 volt for Alexa Fluor 488, 624 volt for PerCP-Cy5-5 and the laser is at 488 nm. In total 50,000 events were collected. The data analysis was done with FlowJo 7.6.5 software (^©^Tree Star, Inc.). First, a singlet cell population was gated through the plots of FSC-W against FSC-H, SSC-W against SSC-H and the histogram of PerCP-Cy5-5 channel for auto-fluorescence from Chlorophyll a and it is usually ~90% of total population. Second, the mean and standard deviation of EYFP emission per cell from singlet cell population were obtained from a histogram of the Alexa Fluor 488 channel.

### Statistical evaluation

The difference between the promoter strengths indicated, reported as fold (±s.e.m.) were tested with the unpaired *t*-test with one-tail (α = 0.05) and n > 45,000. Indicated comparisons are all significantly different.

### The simulation of spontaneous thermal openings of DNA strand

All parameter values used and calculation of thermal opening probability are the same as the Peyrard-Bishop-Dauxois model simulated with Monte Carlo method [26], except the use of temperature 303 K in present study. The potential energy sum of the hydrogen bonding of a base pair and the π-stacking interaction between two adjacent base pairs was simulated to identify the equilibrated configurations of a dsDNA (double-stranded DNA) fluctuating at the temperature 303 K. The spontaneous thermal opening probability was averaged over 2,000 realizations. The simulation algorithm was programed with the Matlab. The 2,000 different seeds of the random number generator, Mersenne Twister, were used for generating pseudorandom numbers in the respective 2,000 realizations of a promoter sequence; keep using this set of seeds to analyze other promoter sequences for comparisons. The simulation was accomplished by submitting the parallel distributed jobs to the cluster computing resource in Uppsala University.

## Competing interests

The authors declare that they have no competing interests.

## Authors’ contributions

HHH and PL conceived of the research; HHH designed the study and performed the experiment; HHH and PL analyzed the data and wrote the manuscript. Both authors read and approved the final manuscript.

## Supplementary Material

Additional file 1 **Supplementary Data for Wide-Dynamic-Range Promoters Engineered for Cyanobacteria.** Contains the table of primers used in the study, the table of measured values in Figure 3, the figure of the weighted sequence logo, the figure of spectra comparison, and the figure of proposed feed-forward loop. Click here for file
