# Supplementary material for: Wide-dynamic-range promoters engineered for cyanobacteria
Source: J Biol Eng. 2013 Apr 22;7:10. doi: 10.1186/1754-1611-7-10 (PMC3724501; doi:10.1186/1754-1611-7-10)
Supplement: Additional file 1 — Supplementary Data for Wide-Dynamic-Range Promoters Engineered for Cyanobacteria. Contains the table of primers used in the study, the table of measured values in Figure 3, the figure of the weighted sequence logo, the figure of spectra comparison, and the figure of proposed feed-forward loop. [file 1754-1611-7-10-S1.pdf]

Supplementary Data for

Wide-Dynamic-Range Promoters Engineered for Cyanobacteria

Hsin-Ho Huang, and Peter Lindblad*

Microbial Chemistry, Department of Chemistry - Ångström Laboratory, Uppsala University, P.O. Box 523, SE-75120, Uppsala, Sweden

* Corresponding author

Email addresses:
HHH: hsin-ho.huang@kemi.uu.se
PL: peter.lindblad@kemi.uu.se

Supplementary Table S1. Primers used in the present study

Primer	Sequence, 5’→3’	note
XhoI_f_pSB2K3	TGCTCGAGGCCGCGATTAAATTCCAACATGGATGCT	for all L promoters
R40c10A-6C_G-5C	GATAGATATAATGCCCACTACTAGTAGCGGCCGCTGCAGTCC	for the L01 promoter
R40c10A-6T_G-5C	GATAGATATAATGTCCACTACTAGTAGCGGCCGCTGCAGTCC	for the L02 promoter
R40c10A-6G_G-5C	GATAGATATAATGGCCACTACTAGTAGCGGCCGCTGCAGTCC	for the L03 promoter
R40c10G-5C	GATAGATATAATGACCACTACTAGTAGCGGCCGCTGCAGTCC	for the L04 promoter
R40c10A-6C_G-5T	GATAGATATAATGCTCACTACTAGTAGCGGCCGCTGCAGTCC	for the L05 promoter
R40c10A-6T_G-5T	GATAGATATAATGTTCACTACTAGTAGCGGCCGCTGCAGTCC	for the L06 promoter
R40c10A-6G_G-5T	GATAGATATAATGGTCACTACTAGTAGCGGCCGCTGCAGTCC	for the L07 promoter
R40c10G-5T	GATAGATATAATGATCACTACTAGTAGCGGCCGCTGCAGTCC	for the L08 promoter
R40c10A-6C	GATAGATATAATGCGCACTACTAGTAGCGGCCGCTGCAGTCC	for the L09 promoter
R40c10A-6T	GATAGATATAATGTGCACTACTAGTAGCGGCCGCTGCAGTCC	for the L10 promoter
R40c10A-6G	GATAGATATAATGGGCACTACTAGTAGCGGCCGCTGCAGTCC	for the L11 promoter
R40c10	GATAGATATAATGAGCACTACTAGTAGCGGCCGCTGCAGTCC	for the L12 promoter
R40c10A-6C_G-5A	GATAGATATAATGCACACTACTAGTAGCGGCCGCTGCAGTCC	for the L13 promoter
R40c10A-6T_G-5A	GATAGATATAATGTACACTACTAGTAGCGGCCGCTGCAGTCC	for the L14 promoter
R40c10A-6G_G-5A	GATAGATATAATGGACACTACTAGTAGCGGCCGCTGCAGTCC	for the L15 promoter
R40c10G-5A	GATAGATATAATGAACACTACTAGTAGCGGCCGCTGCAGTCC	for the L16 promoter
R40c10_GGGA	GATAGATATAATGGGAGCTACTAGTAGCGGCCGCTGCAGTCC	for the L21 promoter
R40_GGGA	TGGGAGCTACTAGTAGCGGCCGCTGCAGTCC	for the L22 promoter
R40c10withTrcDIS	GATAGATATAATGTGTGGTACTAGTAGCGGCCGCTGCAGTCC	for the L31 promoter
dtetLVA_F	TGTGAAAGTGGGTCCTAATAAAACGACGAAAACTAC	LVA tag removal
dtetLVA_R	GTAGTTTTCGTCGTTTTATTAGGACCCACTTTCACA	LVA tag removal
GSP1	AGGGTGTCGCCCT	5’-RACE
GSP2	CGAACTTCACCTCGGCGCGGGTC	5’-RACE
nestGSP	TTGTAGTTGCCGTCGTCCTTGAAGAAGAT	5’-RACE

Supplementary Table S2. The induction fold of L03 promoter in cells of the cyanobacterium *Synechocystis* PCC6803 under different growth conditions

Time (h)	aTc (ng/mL)	light-activated darkness		red light		white light	
		0 mM Glc	5 mM Glc	0 mM Glc	5 mM Glc	0 mM Glc	5 mM Glc
24	0	1 (23±1)[a]	1 (37±3)	1 (31±2)	1 (66±4)	1 (26±1)	1 (28±4)
	10^2	22±1	155±11	87±5	63±5	4.1±0.1	4±1
	10^3	33±1	164±11	134±7	94±6	9.7±0.3	16±4
	10^4	41±1	**239±16**	133±7	97±7	49±15	131±38
48	0	1 (76±46)	1 (63±3)	1 (13±4)	1 (66±13)	1 (29±11)	1 (46±5)
	10^2	11±7	146±8	97±30	32±6	3±1	2.3±0.3
	10^3	16±10	164±9	169±52	65±13	5±2	7±1
	10^4	21±13	143±8	**290±93**	94±20	35±19	50±13
72	0	1 (44±14)	1 (70±4)	1 (9±8)	1 (40±19)	1 (17±16)	1 (15±13)
	10^2	25±8	135±8	85±84	28±13	4±4	2±2
	10^3	36±11	160±10	152±150	56±26	9±8	10±9
	10^4	47±14	161±15	255±254	81±38	35±36	82±72

[a], At an aTc concentration of 0 ng/mL, the value in parentheses is the experimental mean ± s.e.m. of EYFP emission per cell after subtracting the auto-fluorescence of *Synechocystis* cells containing pPMQAK1 vector only. This value is as the denominator to calculate the induction fold when the aTc concentration is 10^2, 10^3, or 10^4 ng/mL in the respective growth condition. The induction fold is presented as mean ± s.e.m.; maximal experimental induction fold in bold. The measurement was done using a flow cytometer to collect 50,000 events for each of the three biological repeats.

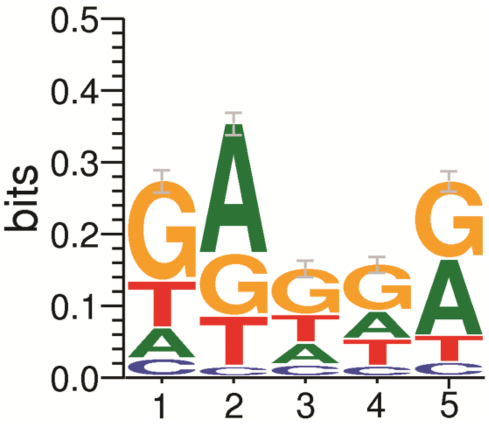

Supplementary Figure S1 The weighted sequence logo of the -10 element downstream sequence of a type I promoters in *Synechocystis* PCC 6803 (*Synechocystis*). The non-template strand sequences between the TSS and the -10 element of a type I promoters of *Synechocystis* were accessed and weighted with the respective “sequence reads”, resulting in a sequence pool, according to the Dataset S1, Table S3 in the work of Mitschke J, *et al*. [25]. The sequence conservation in the region of 5 nt immediately downstream of the -10 element was evaluated together with this sequence pool in bits of information using Weblogo 3.0.

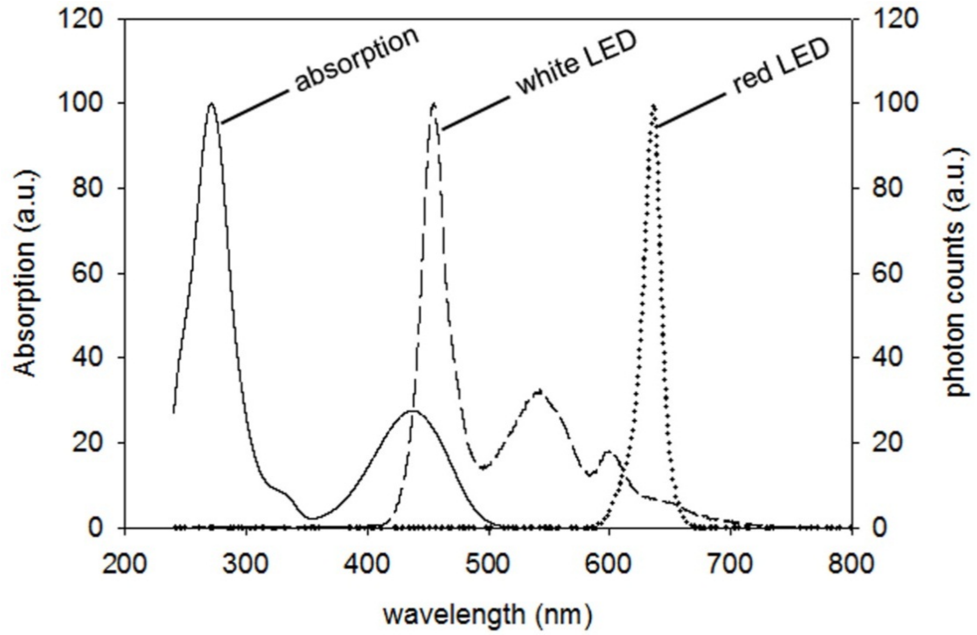

Supplementary Figure S2 The absorption spectrum (solid line) of anhydrotetracycline (aTc) in BG11 medium supplemented with kanamycin and the spectra of photons emitted from white LED (dash line) and from red LED (dot line). The former was acquired by a Cary 5000 UV-VIS-NIR spectrophotometer (Varian) and the latter was recorded using a CCD spectrometer Avantes *AvaSpec-2048* (Azpect Photonics AB).

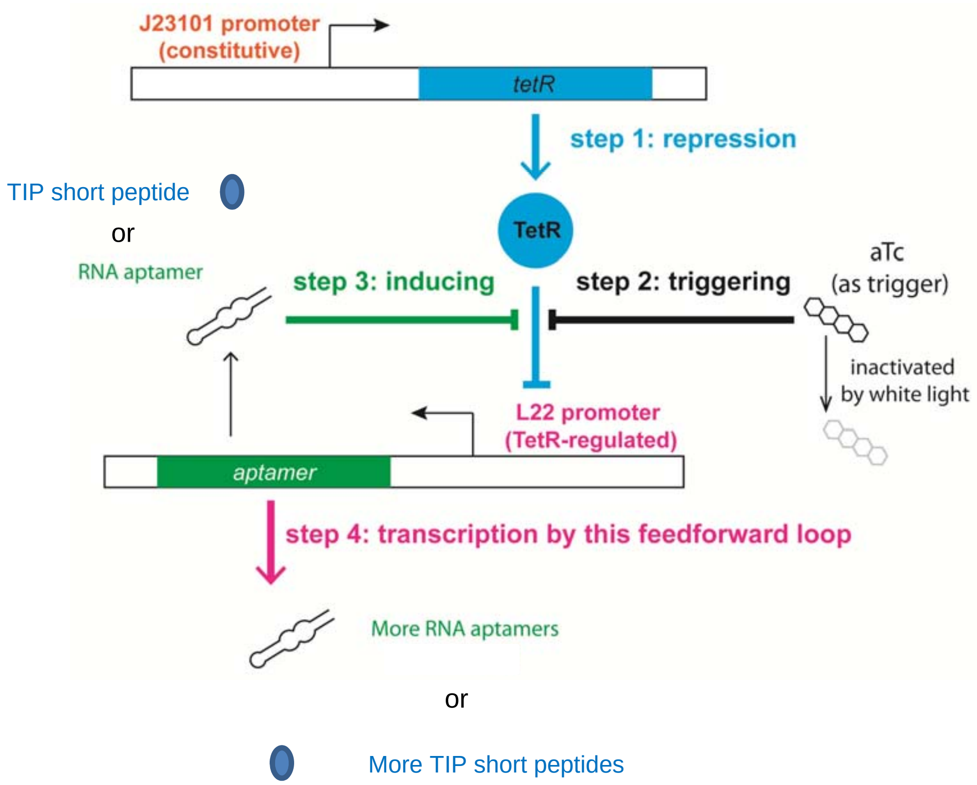

Supplementary Figure S3 The proposed lasting induction of TetR-regulated promoters by a feed-forward loop triggered by aTc. The constitutively-expressed TetR repressors fully repress the L22 promoter (step 1). The aTc triggers the transcription of the RNA aptamer [37] or the expression of the short peptide TIP [38] (step 2). The RNA aptamer or the short peptide TIP auto-induces its transcription (step 3). More RNA aptamers or short peptides are transcribed in this feed-forward loop for inducing other TetR-regulated promoters (step 4).
